# Perceived Behavioral Changes in Early Multiple Sclerosis

**DOI:** 10.1155/2007/674075

**Published:** 2007-05-30

**Authors:** Fabiana Souza Lima, Samanta Simioni, Laure Bruggimann, Christiane Ruffieux, Jean Dudler, Christian Felley, Pierre Michetti, Jean-Marie Annoni, Myriam Schluep

**Affiliations:** ^1^Department of NeurologyCentre Hospitalier Universitaire VaudoisLausanneSwitzerland; ^2^Social and Preventive MedicineCentre Hospitalier Universitaire VaudoisLausanneSwitzerland; ^3^Department of RheumatologyCentre Hospitalier Universitaire VaudoisLausanneSwitzerland; ^4^Department of GastroenterologyCentre Hospitalier Universitaire VaudoisLausanneSwitzerland; ^5^Department of NeurologyGeneva University HospitalSwitzerland

**Keywords:** Multiple sclerosis, behavioral changes, Iowa Scale of Personality Change, Dysexecutive Questionnaire

## Abstract

Acquired behavioral changes have essentially been described in advanced multiple sclerosis (MS). The present study was designed to determine whether behavioral modifications specifically related to the MS pathological process could be identified in the initial phase of the disease, as compared to control patients with chronic, relapsing and progressive inflammatory disorders not involving the central nervous system (CNS). Eighty-eight early MS patients (Expanded Disability Status Scale score ≤ 2.5) and 48 controls were tested. Perceived changes by informants in behavioral control, goal-directed behavior, decision making, emotional expression, insight and interpersonal relationships were assessed using the Iowa Scale of Personality Change (ISPC). Executive behavioral disturbances were screened using the Dysexecutive Questionnaire (DEX). The mean change between the premorbid and postmorbid ISPC ratings was similar in the MS [12.2 (SD 15.6)] and in the control [11.5 (SD 15.1)] group. The perceived behavioral changes (PBCs) most frequently reported in both groups were *lack of stamina*, *lability/moodiness*, *anxiety*, *vulnerability to stress* and *irritability*. Pathological scores in the DEX were also similar in both groups. Correlations between PBCs and DEX scores were different in MS and control groups. MS patients with cognitive impairment had a marginally higher number of PBCs than control patients (*p* = 0.056) and a significantly higher DEXp score (*p* = 0.04). These results suggest that (1) PBCs occurring in early MS patients were not different from those induced by comparable chronic non-CNS disorders, (2) qualitative differences in the relationship between behavioral symptoms and executive-behavioral changes may exist between MS and control groups, and (3) behavioral symptoms seem associated with cognitive deficits in MS. We further plan to assess these observations longitudinally.

## 1. Introduction

Cognitive dysfunction has been well documented in multiple sclerosis (MS) and occurs in up to 60% of MS patients [14,18]. However, the behavioral and affective impairments linked to MS have not been systematically investigated, especially in early MS, and are generally not part of the health status assessment of MS patients. Behavioral manifestations, classically described as a variety of mood-related symptoms, such as cheerfulness, optimism, denial of disability, and pathological laughing, were once considered the hallmark of MS but are, in fact, rather rare [19]. Such behavioral alterations are thought to represent more an associated feature of MS induced central nervous system (CNS) pathology, rather than a maladaptive response of the subjects and are, consequently, prone to remain stable. In particular, euphoric mood aberrations seem to be

[1]Authors Fabiana Souza Lima and Samanta Simioni contributed equally to the present work.

ISSN 0953-4180/07/$17.00 © 2007 – IOS Press and the authors. All rights reserved substrate-dependent disturbances and tend to evolve in parallel with disease duration and severity [7]. Other behavioral changes, such as agitation, irritability, apathy and disinhibition, are frequent [6] and seem to play an important role in MS [5]. It is therefore reasonable to hypothesize that behavioral changes are present early in MS and may not necessarily be linked to purely cognitive or adaptative parameters.

While the above studies suggest that, in advanced MS, behavioral changes are consistent with the acquired neurological dysfunction, this is still debatable in the initial phase of the disease. In the early phase, such modifications may be due to two different mechanisms: (i) alterations of cortical and subcortical circuits involving the orbitofrontal, medial and dorsolateral prefrontal cortex [21], which modify patients' behavior [12] or their ability to verbalize the emotional status [16], or (ii) maladapted coping responses, which may occur during any chronic illness (also non neurological) characterized by an unpredictable functional decline, that may have clinically relevant psychological implications [1,8]: a chronic disease such as MS, may represent a challenge to face, and patients' affective reactions, depending on their personality and cognition, could then play a role in their adaptative (or maladaptive) behavior. Distinguishing between these two mechanisms is important, as it may influence the type of therapeutic approach that is indicated.

This study was designed to evaluate behavioral changes in a homogeneous cohort of early MS patients with minor impairment in parallel to a comparable cohort of control patients with chronic, relapsing, and progressive disorders not involving the CNS. Behavioral modifications occurring in neurological diseases have recently been approached by the mean of scales which measure changes perceived by patients' informant [2], and initially employed for the assessment of traumatic brain injury patients. These scales represent an advance in the quantitative measurement of specific behavioral and behavioral-executive symptoms and have been used in different neurological conditions. We hypothesized that such changes occur in early MS and questioned whether they are MS-specific. Changes specific to MS would be a sign that behavioral changes early in the course of the disease could be due to preclinical subcortical dysfunction, whereas the occurrence of identical behavioral changes both in diseased controls and MS patients would argue that early behavioral modifications are related to a non-specific psychological reaction to sickness.

## 2. Materials and methods

### *2.1. MS and control patients*

Eighty-eight patients with a diagnosis of MS or possible MS based on McDonald criteria [15,17] were tested. Of these, 51 had relapsing-remitting (RR) MS, another 13 with confirmed MS had experienced a single relapse and showed dissemination in time and space proven by magnetic resonance imaging (MRI), and 24 patients had possible MS with a single relapse, positive oligoclonal bands in the cerebrospinal fluid, and a positive MRI at the time of diagnosis. Ten of these 24 patients developed a second relapse during follow-up, confirming the diagnosis of MS (mean interval to diagnosis confirmation: 11.7 months, SD 8.8).

The criteria used to define our early MS population were: (i) an Expanded Disability Status Scale (EDSS) [13] score $\leqslant$ 2.5, (ii) no or little handicap for vocational and leisure activities measured on the London Handicap Scale [11], then transposed to a validated functional score ranging from 1 to 4 according to the classification used in the global functional status in rheumatoid arthritis (see Table 1) [9], (iii) MS duration $>$3 months and $\leqslant$ 5 years. Medication, in particular interferon-$\beta$1a or 1b and antidepressants, was not considered an exclusion criterion. All MS patients were evaluated for long-term memory (Rey's Auditory Verbal Learning Test), executive functions (Behavioural Assessment of the Dysexecutive Syndrome) and attention (Trail Making Test) [12]. Cognitive impairment was defined by a performance two standard deviations (SD) below the appropriate mean for a given test. Fatigue was assessed using the severity subscore of the Fatigue Assessment Instrument (FAI) [20] (cut-off value $<$ 4.89).

Forty-eight control patients without CNS involvement and comparable in terms of age, education, and handicap were recruited. These patients suffered from systemic inflammatory disorders that, like MS, evolve chronically with exacerbations: rheumatoid arthritis ($n$ = 11), ankylosing spondylarthritis ($n$ = 13), psoriatic arthritis ($n$ = 1), motor multifocal neuropathy ($n$ = 2), and inflammatory bowel disease ($n$ = 21). All participating control patients had no or only a minor handicap for vocational and leisure activities according to the classification of functional status in rheumatoid arthritis [9], and a disease duration of $>$3 months and $\leqslant$ 10 years (a higher upper limit than that used for the MS patients was chosen to obtain a large enough number of controls because of the longer time needed to

Table 1
Demographic data for the MS and control patients

| | MS patients ($n = 74$) | Control patients ($n = 48$) |
|---|---|---|
| Female gender [n (%)] | 50 (67.6) | 24 (50) |
| Age (years) [mean (SD)] | 34.1 (9.9) | 31.5 (8.9) |
| $<$ 9 years (%) | 13.5 | 25.0 |
| Schooling 9–12 years (%) | 59.5 | 45.8 |
| $>$ 12 years (%) | 27.0 | 29.2 |
| Disease duration (years) [mean (SD)] | 2.5 (1.7) | 5.0 (3.0) |
| Handicap[a] [mean (SD)] | 1.7 (0.4) (class 1: $n = 36$; class 2: $n = 23$; class 3: $n = 15$) | 1.9 (0.7) (class 1: $n = 14$; class 2: $n = 27$; class 3: $n = 7$) |
| Immunomodulatory therapy [n (%)] | 32 (46.4) | 20 (45.5) |
| Antidepressants [n (%)] | 5 (6.7) | 2 (4.2) |

[a]Handicap rated according to the functional scale used in rheumatoid arthritis [9] as: class 1 = able to perform usual activities of daily living (self-care, vocational, and avocational); class 2 = able to perform usual self-care and vocational activities, but limited in avocational activities; class 3 = able to perform usual self-care activities, but limited in vocational and avocational activities; class 4 = limited in ability to perform usual self-care, vocational, and avocational activities.

develop an equivalent handicap in these disorders). Immunomodulatory, immunosuppressive and antidepressive treatments were permitted. Since they were young adults with no CNS pathology, the controls were not assessed for cognition.

MS and control patients presenting psychiatric disorders diagnosed according to the DSM-IV criteria were not excluded. Inclusions were performed at distance from exacerbations or corticosteroid treatment ($\geqslant$ 6 weeks). All patients signed an informed consent form before participating in this study, which had been approved by the local Ethical Committee.

### 2.2. Scales measuring behavioral changes

The Iowa Scale of Personality Change (ISPC) [2] provides, through 26 behavioral symptoms related to maladaptive functioning, an exhaustive checklist of the modifications that can occur after brain lesions in the affective, behavioral and social dimensions, and measures the extent of the change that an informant perceives in the patients' behavior in comparison to his/her premorbid status (Fig. 1). This scale has been validated for patients' informants in a wide range of neurological disorders, and was chosen as a useful tool in previous studies focused on perceived behavioral changes in different neurological conditions [3,10]. The information is provided by a close relative who was familiar with the patient before the onset of the disease. Two global scores are obtained from the sum of the ratings for all items, the first reflecting the patient's perceived behavior during the six months period before disease onset (premorbid score) and the second corresponding to the patient's perceived behavior at inclusion in the study (postmorbid score) [3]. The difference between these two scores provides a measure of the perceived extent of the changes (differential score). The ISPC includes also three characteristics (vanity, frugality and manipulativeness), not found in acquired brain damage and examined to detect possible response biases. A change of $>$3 in any of these three "control" items invalided the patient's rating. A second measurement was to detect which behavioral symptom was significantly increased or decreased (defined by Barrash [3] as *acquired personality traits* [APTs], but that we will rename here *perceived behavioral changes* [PBCs]). PBCs were defined, for each patient, as scores showing at least a moderate disturbance in the postmorbid state (cut-off score $\geqslant$ 4) with a change in score of $\geqslant$ 1 from the premorbid level. The 26 subscales can be grouped into the five main factors of distress, impaired ego functioning, executive dysfunction, interpersonal disturbance, and hypo-emotionality. These factors were also used for analysis.

The Dysexecutive Questionnaire (DEX) [24], one of the few available standardized rating scales, was used for quantifying behavioral disturbances commonly associated with executive impairment. The 20 items of the DEX encompass broad areas of likely changes, which can be grouped into the five factors of inhibition, intentionality, executive memory, positive affect, and negative affect [4]. Each item is scored on a 5-point scale ranging from "never" to "very often" (0 to 4). Unlike the ISPC, the DEX is provided in two versions, DEXp (cut-off value $<$38), designed to be completed by the patient, and DEXr (cut-off value $<$24.5), to be completed by a close relative; both were used for analysis.

| | |
|---|---|
| **Inflexibility** - The extent to which he stubbornly holds onto his attitudes and opinions; and the extent to which he has difficulty trying different approaches to a problem, even if his way isn't working. | |
| *Which rating best characterizes the way he is now?* | |
| 1. | He is quite open-minded, and will consider several sides of an issue before making up his mind. He is quite open to suggestions about a better way to do something. |
| 2. | |
| 3. | He listens to and thinks about other people's points of view, even though he has his own opinion on most subjects. Although he usually has his preferred way of doing something, it usually doesn't bother him to listen to the suggestions of others. If the way he is going about something isn't working, he is usually willing to try a new approach. |
| 4. | |
| 5. | He has a pretty strong opinion about most things. It is very difficult to get him to change his mind without clear proof, and his stubbornness sometimes irritates friends or family. He frequently argues over insignificant matters. He usually thinks his way of doing something is the best way, and he doesn't like others telling him how things should be done. Sometimes he is slow to realize that the way he has been going about something is not going to work. Even after it should be clear that his way is not working out, he will often make several more efforts before he will try a new approach to the problem, or will listen to suggestions. |
| 6. | |
| 7. | He is so stubborn that friends and family often get very frustrated or irritated with him. As a result, people who know him often avoid discussing many things with him to avoid arguments. He thinks there is only one way to do things—his way—and he will rarely consider other approaches, even if his way is not working. If his way is not working, he may keep on making the same mistakes over and over. |

Fig. 1. Example of a rating sheet for one item of the ISPC (total number of items: 29). For each item, a rating of 1 indicates excellent, 3 average, 4–5 moderate disturbance, and 6–7 severe disability. Ratings 1, 3, 5, and 7 correspond to brief definitions of the worsening of the different behavioral characteristics, while ratings 2, 4, and 6 reflect intermediate measurements.

### *2.3. Statistical analysis*

We calculated for the three main outcomes and for the actual sample sizes the difference that could be evidenced with a power of 80%. MS patients should have had a mean number of PBCs of 7.1 (SD 5) for a difference could be shown with control patients (mean 4.4, SD 5). For the DEXr score, the test could show a difference between 20.0 and 26.3 (SD = 12) and for the DEXp score, between 23.9 and 30.2 (SD = 12). A Wilcoxon test was used to compare demographical data, handicap, DEX and ISPC scores between the control and MS groups and for subgroup analysis. To address difference between paired measures of ISPC scores (before onset and at inclusion) and of DEX scores (DEXp and DEXr), a paired Wilcoxon test was performed. For the refined analysis of ISPC items, the percentage of patients by group meeting the PBC criteria was calculated for each item. An exploratory analysis was conducted to screen items with a large difference between both groups using independent chi2 tests. Correlations between the DEX and ISPC scores were examined using the Spearman correlation. DEX scores and the number of PBCs were fitted by a MANOVA model including gender, grouping of patients (controls, MS patients without cognitive deficits and with cognitive deficits), and an interaction term. The level for statistical significance was taken as a p-value <0.05.

## 3. Results

Seventy-four of the 88 MS patients tested were retained for analysis, since 14 patients with possible MS did not confirm their diagnosis during the follow-up period determined by the time necessary to complete this study. The demographic data for the MS and control patients are summarized in Table 1. The two groups were comparable in terms of age ($p = 0.1$), education ($p = 0.1$), handicap ($p = 0.1$), and immunomodula-

Table 2
Values for, and difference between, the global ISPC scores in the MS and control patients

| | MS patients ($n = 69$) Mean (SD) | Control patients ($n = 44$) Mean (SD) | $p$ value |
|---|---|---|---|
| Premorbid ISPC | 67.3 (14.7) | 64.0 (10.7) | *0.3* |
| Postmorbid ISPC | 79.5 (17.8) | 75.4 (18.0) | *0.2* |
| Difference | 12.2 (15.6) | 11.5 (15.1) | *0.7* |
| $p$value | $< 0.001$ | $< 0.001$ | |

tory therapy ($p = 0.9$). Marginal gender effect ($p = 0.05$) reflects the female overrepresentation classically observed in MS population. Twenty-five MS patients (33.8%) had cognitive deficits. Executive impairment, known to be associated with certain behavioral changes, was seen in five patients. Pathological fatigue symptoms were reported by 54.4% of the MS patients. Finally, 4 MS patients had a diagnosis of depression according to the DSM-IV criteria and 5 were receiving antidepressants.

### *3.1. ISPC scores in the MS and control groups*

Five MS patients and 4 controls were excluded from the analysis because of missing values or of a differential score of >3 for one of the "control" items. The average global ISPC score, thus based on 69 MS patients and 44 controls, was in the MS group 67.3 (SD 14.7) before MS onset and 79.5 (SD 17.8) at inclusion, the respective corresponding values for the control group being 64.0 (SD 10.7) and 75.4 (SD 18.0) (Table 2). The two groups were similar in terms of premorbid score ($p = 0.3$) and postmorbid score ($p = 0.2$). The mean difference between the premorbid and postmorbid ratings was 12.2 (SD 15.6) in the MS group and 11.5 (SD 15.1) in the control group. There was no difference between the groups in terms of the extent of the changes ($p = 0.7$) but postmorbid scores were indeed statistically different from premorbid scores in both groups ($p < 0.001$ in both groups).

### *3.2. Frequency of PBCs in MS and control patients: item by item analysis*

The MS and control groups were compared by evaluating the number of items satisfying the conditions for a PBC (postmorbid score $\geqslant 4$, difference $\geqslant 1$). The average number of PBCs was 4.8 (SD 5.2) in the MS group and 4.4 (SD 5.0) in the control group. The Wilcoxon test showed no difference between the groups in the distribution of the number of PBCs ($p = 0.7$). The percentage of patients in both groups meeting the PBC criteria for each item is shown in Table 3. Five items were considered as PBCs in more than 30% of MS patients; all five also met the PBC criteria in more than 25% of the controls. More than 50% of MS patients were perceived by their relatives as having changed on the item *lack of stamina*, 40% on the item *lability/moodiness*, and more than 30% were perceived as more irritable, anxious, and vulnerable to stress. Ranking the items according to the frequency of patients being perceived by their relative as having changed issued in a quite similar order in both groups (Spearman's rho = 0.72). The item *inflexibility*, present in 26.1% of MS patients vs. 9.1% of controls ($p = 0.02$) showed discrepancy. The item *lack of stamina* was significantly higher ($p = 0.002$) in MS patients with a significant fatigue score (70.2%) than in those with a low score of fatigue (34.2%).

### *3.3. Subgroup analysis of the number of PBCs*

A subgroup analysis was done on the basis of gender, disease duration, immunomodulatory treatment, and cognitive status (this last factor concerning only MS group). The average number of PBCs was higher in women (6.2, SD 5.4) than in men (2.6, SD 3.9) in the control group ($p = 0.01$), but not in the MS group (4.9, SD 5.5 vs. 4.6, SD 4.4, respectively; $p = 0.9$). A subgroup analysis for gender showed no difference in the number of PBCs between controls and MS patients ($p = 0.1$ for both gender). Comparing the number of perceived behavioral changes between patients divided into subgroups according to disease duration ($< 2.5$ years vs. $> 2.5$ years), no significant difference was shown either in the controls (mean 6.0, SD 3.9 vs. mean 4.0, SD 5.2; $p = 0.06$) or the MS group (mean 5.5, SD 5.8 vs. mean 3.9, SD 4.1; $p = 0.4$). Immunomodulatory therapy was not associated to an increased number of PBCs in the treated (mean 3.9, SD 5.3) vs. non-treated (mean 4.8, SD 4.8) control subgroups ($p = 0.4$). In the MS patients, the number of PBCs was 5.8 (SD 4.8) in the treated and 4.0 (SD 5.4) in the non-treated subgroup ($p = 0.06$).

Concerning cognition, the intragroup analysis revealed that the mean number of PBCs differed signifi-

Table 3
Percentage of MS and control patients showing a PBC for each of the 26 items of the ISPC

| ISPC scale | MS patients (%) | Control patients (%) |
|---|---|---|
| Lack of stamina | **52.2** | **43.2** |
| Lability/moodiness | **40.6** | **34.1** |
| Irritability | **33.3** | **31.8** |
| Anxiety | **31.9** | **40.9** |
| Vulnerability to stress | **31.9** | **27.3** |
| Depression | 27.5 | 25.0 |
| Inflexibility | 26.1 | 9.1 |
| Impatience | 21.7 | 27.3 |
| Social withdrawal | 21.7 | 9.1 |
| Apathy | 18.8 | 11.4 |
| Obsessiveness | 15.5 | 11.4 |
| Indecisiveness | 15.9 | 13.6 |
| Dependency | 15.9 | 18.2 |
| Insensitivity | 14.5 | 13.6 |
| Lack of initiative | 14.5 | 18.2 |
| Suspiciousness | 13.0 | 15.9 |
| Impulsivity | 11.6 | 9.1 |
| Lack of persistence | 10.1 | 11.4 |
| Lack of insight | 10.1 | 4.6 |
| Perseveration | 10.1 | 13.6 |
| Type A behavior | 10.0 | 11.9 |
| Blunted emotional experience | 8.7 | 13.6 |
| Lack of planning | 8.7 | 20.5 |
| Aggression | 8.7 | 2.3 |
| Poor judgment | 8.7 | 6.8 |
| Inappropriate affect/emotion | 7.2 | 4.6 |
| Social inappropriateness | 2.9 | 4.6 |

Table 4
Values for, and difference between, the two DEX total scores

| | MS patients ($n = 73$) Mean (SD) | Control patients ($n = 47$) Mean (SD) | $p$ value |
|---|---|---|---|
| DEXp | 24.6 (11.7) | 23.9 (12.4) | *0.7* |
| DEXr | 20.9 (13.7) | 20.0 (11.4) | *0.9* |
| Difference | 3.7 (9.8) | 3.9 (8.6) | *0.9* |
| $p$ value | *0.003* | *0.008* | |

cantly ($p = 0.005$) between MS patients with cognitive deficits (mean 7.7, SD 6.8) and those without (mean 3.4, SD 3.5). A subsequent analysis showed that the 25 cognitively impaired MS patients had a marginally higher number of PBCs than control patients ($p = 0.056$).

### 3.4. DEX global scores

One MS patient and one control were excluded from the analysis because of missing values. Using the DEXr [24], 35.6% of MS patients and 25.5% of controls had a significant pathological score although both groups did not differ significantly ($p = 0.2$). The scores for DEXp, DEXr and the difference between the two (DEXp-DEXr) were comparable in both groups (Table 4). Relatives' ratings were significantly lower than patients' ratings in both groups (MS group: $p = 0.003$; control group: $p = 0.008$). The Spearman correlations of DEXp and DEXr were 0.59 (95% CI: 0.40,0.73) in the MS group and 0.65 (95%CI: 0.43,0.80) in the control group.

### 3.5. DEXr subgroup analysis

Subgroup analysis for gender showed that DEXr score was significantly higher for women (mean 24.6, SD 11.6) than for men (mean 15.9, SD 9.5) in the control group ($p = 0.005$). In the MS group, DEXr was lower for women (mean 18.6, SD 11.7) than for men (mean 25.9, SD 16.6) but the difference was not significant ($p = 0.08$). A significant difference between MS and control patients was shown for men ($p = 0.03$) and women ($p = 0.04$). No association was found be-

Table 5
Spearman correlations between selected corresponding ISPC (postmorbid scores) and DEXr items

| ISPC items | DEX items | MS | 95%CI | Control | 95%CI |
|---|---|---|---|---|---|
| Impulsivity | Item 2 | 0.39 | 0.16,0.58 | 0.68 | 0.47,0.82 |
| Lack planning | Item 4 | 0.23 | −0.1,0.45 | 0.32 | 0.02,0.57 |
| Irritability | Item 5 | 0.56 | 0.37,0.71 | 0.46 | 0.18,0.67 |
| | Item 12 | 0.47 | 0.26,0.64 | 0.48 | 0.20,0.69 |
| Lack insight | Item 7 | 0.23 | −0.01,0.45 | 0.16 | −0.15,0.44 |
| Apathy | Item 8 | 0.44 | 0.22,0.62 | 0.41 | 0.12,0.64 |
| Social inappropriateness | Item 9 | 0.27 | 0.03,0.48 | 0.40 | 0.11,0.63 |
| | Item 20 | 0.35 | 0.12,0.55 | −0.12 | −0.41,0.19 |
| | Item 13 | 0.49 | 0.28,0.66 | 0.33 | 0.03,0.58 |
| Blunted emotional experience | Item 11 | 0.27 | 0.03,0.48 | −0.05 | −0.35,0.26 |
| Perseveration | Item 14 | −0.11 | −0.34,0.14 | 0.05 | −0.26,0.35 |
| | Item 16 | 0.01 | −0.23,0.25 | 0.22 | −0.09,0.49 |
| Lack of persistance | Item 18 | 0.43 | 0.21,0.61 | 0.30 | −0.01,0.56 |

tween DEXr scores and disease duration (cut-off 2.5 years) either in MS (mean 20.0, SD 11.4 vs. mean 17.9 SD 10.8; $p = 0.2$) or for controls (mean 21.4, SD 6.7 vs. mean 19.8 SD, 12.4; $p = 0.9$). Patients receiving immunomodulatory or immunosuppressive treatments were also similar to untreated patients in MS patients ($p = 0.3$) and in control diseases ($p = 0.7$). DEXr scores did not differ between MS patients with and without cognitive impairment (mean 19.1, SD 12.7 vs. mean 24.6, SD 15.3; $p = 0.1$). MS patients with cognitive deficits had DEXr scores similar to control patients ($p = 0.2$).

### 3.6. *DEXp subgroup analysis*

Subgroup analysis for gender showed that DEXp score was marginally higher for women (mean 27.1, SD 13.7) than for men (mean 20.1, SD 9.8) in the control group ($p = 0.056$). In the MS group, there was no gender effect (women: mean 23.8, SD 11.2; men: mean 26.2, SD 12.8; $p = 0.4$). Finally, DEXp score was not significantly different between MS and control patients, neither for women nor for men. No association was found between DEXp scores and the duration of the disease for both groups (MS patients: mean 25.9, SD 10.9 vs. mean 20.9, SD 9.9; $p = 0.2$; controls: mean 22.7, SD 10.1 vs. mean 23.9, SD 13.5; $p = 0.7$). Patients receiving immunomodulatory or immunosuppressive treatments had similar DEXp scores that untreated MS (mean 23.8, SD 10.6 vs. mean 25.2, SD 12.7; $p = 0.7$) and control patients (mean 23.0, SD 9.8 vs. mean 24.2, SD 12.6; $p = 0.9$).

Concerning cognition, the intragroup analysis showed that DEXp score was higher in MS patients with cognitive impairment (mean 29.2, SD 10.7) than in those without (mean 22.3, SD 11.6; $p = 0.009$). A subsequent analysis revealed that MS patients with cognitive deficits had DEXp scores significantly higher than control patients ($p = 0.04$).

### 3.7. *Correlations between DEX and ISPC scores*

A 78% higher correlation was found between the number of PBCs and the DEXr score for the MS group (Spearman's rho = 0.66 (95%CI: 0.50,0.78); $p < 0.001$) than for controls (Spearman's rho = 0.37 (95%CI: 0.07,0.61); $p = 0.01$). A correlation was found between the number of PBCs and the DEXp score for the MS group (Spearman's rho = 0.45 (95%CI: 0.23,0.62); $p < 0.001$) but not for the control group (Spearman's rho = 0.27 (95%CI: −0.04,0.53); $p = 0.07$). Here, the strength of correlation was 66% higher in the MS group than for controls.

Spearman correlation between ISPC dysexecutive factor (postmorbid score) and DEXr total score was 0.52 (95%CI: 0.32,0.68) in the MS group, and 0.36 (95%CI: 0.06,0.60) in the control group, whereas for the DEXp total score it was 0.39 (95%CI: 0.16,0.58) in the MS group and 0.25 (95%CI: −0.06,0.52) in the control group. Correlations between items aimed to measure the same behavioral symptoms in the ISPC and the DEXr scale were satisfying for *irritability*, *apathy*, *social inappropriateness* and *lack of persistance* in both groups (Table 5).

Finally DEXp and DEXr scores, and the number of PBCs were jointly fitted using a multiple analysis of variance with gender, grouping of patients (controls, MS patients without cognitive deficits and with cognitive deficits), and an interaction term for gender and the 3 groups. Adjusted estimated means and 95% confidence interval are shown in Table 6. The interaction term was significant ($p = 0.001$).

Table 6
Estimated mean of DEX scores and the number of PBCs with 95% confidence intervals adjusted for MS (with [ci$^-$] and without [ci$^+$] cognitive impairment) and control groups and gender

| | Men | | | Women | | |
|---|---|---|---|---|---|---|
| Patients | MS [ci$^+$] | MS [ci$^-$] | Control | MS [ci$^+$] | MS [ci$^-$] | Control |
| DEXp | 31.0 | 20.2 | 19.2 | 25.6 | 22.7 | 27.8 |
| | (22.6,39.4) | (14.1,26.4) | (14.3,24.1) | (19.7,31.5) | (18.9,26.5) | (23.0,32.6) |
| DEXr | 28.3 | 17.4 | 13.9 | 17.2 | 18.6 | 25.7 |
| | (20.4,36.2) | (11.6,23.2) | (9.3,18.5) | (11.6,22.8) | (15.1,22.2) | (21.2, 30.2) |
| Number of PBCs | 7.4 | 2.7 | 2.8 | 7.9 | 3.6 | 6.2 |
| | (3.8,11.0) | (0.0,5.3) | (0.6,4.9) | (5.4,10.5) | (2.0,5.3) | (4.0, 8.3) |

## 4. Discussion

PBCs were assessed simultaneously in early MS patients and in a comparable chronic non-CNS inflammatory disease group with no more than minimal functional impairment. The main results coming out of our analyses were the followings: i) global DEX scores showed that patients did not underestimate their behavioral modifications compared to informants, thus arguing against anosognosia in the early phase of MS, such as in the control group; ii) informants perceived similar behavioral changes in both MS and control groups, arguing against a pattern of behavioral changes specific for early MS; iii) however, differences were observed in the relationship between the number of PBCs and DEX scores in MS and control patients; moreover, the subgroup of cognitively impaired MS patients tended to have more behavioral changes than controls.

The DEX scores provided by the patients and their relatives were slightly but significantly higher in patients in both groups, suggesting that patients tended to over-evaluate their behavioral changes compared to their relatives in the perception of the executive behaviors. This pattern is unusual in neurological pathologies, where patients after focal lesions or diffuse lesions tend to underestimate their difficulties [23]. A limitation could be the cross-sectional design of this study and such data should be confronted with a longitudinal follow-up. However, the present results may give indices for the interest of this type of research early in the MS process, while patients are still active and fully ambulatory. These data confirm other studies [5] which demonstrated that behavioral modifications such as apathy, desinhibition and dysexecutive syndrome were perceived more intensely by the patients than the relatives, and argue against the presence of anosognosia in early MS. This is further supported by the direct comparison between DEXp and DEXr reports showing a good consistency of DEX scores obtained from patients and relatives, as well as by the minor modification obtained for the item *lack of insight* in the ISPC.

The results obtained through the ISPC also evidenced the occurrence of perceived behavioral modifications in early MS. Irritability, lack of stamina, anxiety, lability/moodiness, and vulnerability to stress were behavioral symptoms perceived as changed in more than 30% of MS patients. This result is partly due to the fact that, to improve sensitivity, we used a cut-off value (ISPC score $\geqslant$4) one point lower than that used in the original methodology [3]. The use of a unique questionnaire to the informant that does not include presently a complementary version intended to be completed by the patient him/herself prevents controlling for a possible exaggeration or underscoring of the PBCs. However, this possibility is unlikely since the differences we obtained between the DEXr and DEXp scores suggest that early MS patients tend to over-score their behavioral changes. An important result was that the control group had a virtually identical frequency of PBCs than MS patients. The similar profile of changes found in other chronic inflammatory diseases not affecting the CNS questions whether these findings are related to early MS pathology.

On the other hand, some of our results suggest that qualitative behavioral differences exist between MS and control patients. First, the relationship between the number of PBCs and DEX scores was markedly different between MS and control subjects, with a Spearman's rho value in the former group 78% larger than in the controls for the DEXr, and 66% larger for the DEXp. Thus, patients' executive behaviors and informants' PBCs look to be differently related in the two groups of patients. Illustrations of such differences can be found in our correlative analysis between DEXr and ISPC items belonging to the same factors and shown in Table 5: items such as impulsivity, social inappropriateness, or lack of persistence were characterized by very different correlative values in MS and control groups, suggesting that these symptoms may have different consequences in the functioning of these groups. Second, we showed that cognitively impaired MS patients had more behavioral changes (ISPC, DEXp). These data may indicate that in MS patients, cognitive deficits are associated in a non-random way to behavioral changes, although this ought to be developed in a longitudinal study with the specific question of a relation between cognitive and behavioral changes [22]. The observation that MS patients treated with immunomodulators scored slightly higher on the ISPC than untreated MS patients gives a additional weight to the interpretation that a neurological dimension is expressed in the PBCs. MS patients are indeed more likely to be given chronic treatment if they are at higher risk of developing severe MS, as assessed by clinical and laboratory parameters (e.g. relapse rate, relapse recovery, EDSS changes, and lesion load and activity on the brain MRI), suggesting that this "at risk" subgroup of MS patients could show more affective changes.

In conclusion, this cross-sectional study is in favor of the presence of similar behavioral modifications both in MS and in other non CNS chronic disorders, and gives no strong argument for the detection of an MS-specific behavioral profile in the early stage of this disease. However, the qualitative differences in MS and control patients concerning the relationship between executive-behavior and PBCs, as well as the association observed between cognitive deficits and the occurrence of PBCs in the MS group, suggest a neurological origin and need further investigations to better understand its specificity for MS pathology. These variations may represent the first signs of a delayed behavioral change occurring in more advanced MS and related to a greater extent of the disease. Nevertheless, this study has some methodological limitations related to missing informations on the reliability of the scales used, to the unbalanced size of the two groups of patients who were not matched for gender (this being related to the female overrepresentation usually observed in any MS population), and finally to the bias introduced by any cross-sectional design evaluating behavioral changes. For these reasons, the present MS cohort will be evaluated longitudinally in the aim to determine which behavioral traits develop further during the course of MS, and are thus specific manifestations of the MS process.

## Acknowledgments

The authors are grateful to Ms G. Le Goff (specialized MS nurse) for her important contribution to the study, and to Ms C. Peter-Favre and A.-C. Juillerat for providing access to the Iowa French version. This work was supported by grants from the Swiss Society for Multiple Sclerosis (MS) and the Swiss National Science Foundation (3200BO-104051, MS). Dr F. Souza Lima was supported by a grant from the European Association of Young Neurologists and Trainees.
